# Systemic Surveillance Guidelines for Uveal Melanoma: A Systematic Review

**DOI:** 10.1111/ceo.70002

**Published:** 2025-09-24

**Authors:** Farzana Y. Zaman, Aisha Ghaus, Mark Shackleton, Damien Kee, Anthony M. Joshua, Roderick O'Day, Malaka Ameratunga

**Affiliations:** ^1^ Department of Medical Oncology Alfred Health Melbourne Victoria Australia; ^2^ School of Translational Medicine Monash University Melbourne Victoria Australia; ^3^ Department of Medical Oncology Olivia Newton‐John Cancer Centre, Austin Health Melbourne Victoria Australia; ^4^ Department of Medical Oncology Peter MacCallum Cancer Centre Melbourne Victoria Australia; ^5^ Kinghorn Cancer Centre St Vincent's Hospital/Garvan Institute of Medical Research Sydney New South Wales Australia; ^6^ School of Clinical Medicine, UNSW Medicine and Health, St Vincent's Healthcare Clinical Campus, Faculty of Medicine and Health UNSW Sydney Sydney, New South Wales Australia; ^7^ Department of Ocular Oncology Royal Victorian Eye and Ear Hospital Melbourne Victoria Australia

**Keywords:** choroidal neoplasm, recurrence, surveillance, uveal melanoma

## Abstract

**Background:**

Uveal melanoma (UM) is the most common primary intraocular tumour. Despite effective local therapies, UM has a high risk of metastatic recurrence, most frequently to the liver. A significant proportion of patients treated definitively for primary UM eventually experience metastatic disease. Systemic surveillance to detect recurrence is critical to maximise therapeutic options. Whilst international guidelines exist, there are currently no standardised Australian guidelines for surveillance imaging. This systematic review examines the literature regarding systemic surveillance methods following local treatment for UM.

**Methods:**

Medline, Embase and PubMed databases were searched, from 2010 to 01‐07‐2024, using keywords related to uveal melanoma and surveillance. Eligible studies were identified by two independent reviewers, and a systematic review was undertaken.

**Results:**

Of 840 records, six guidelines and institutional consensus statements were identified, and an additional 13 studies were included. Most studies were cohort studies (*n* = 7), with the rest being case–control studies and reliability analyses. Risk stratification methods and surveillance strategies varied, with most studies recommending increased frequency (at least every 6 months) and higher‐resolution imaging modalities (MRI over ultrasound) for higher‐risk patients.

**Conclusion:**

Despite several published guidelines, existing evidence regarding optimal surveillance strategies in localised primary UM is of variable quality, relying on cohort studies and limited by heterogeneity, as assessed by the modified Newcastle‐Ottawa Scale. There is a clear need to further define local practices and outcomes to direct future guidelines.

## 1 Introduction

Uveal melanoma (UM) is the most common primary intraocular tumour in adults. In Australia, between 1982 and 2014, there were 4617 cases of UM, with an average age‐standardised incidence rate of 7.6 per million [1]. Standard local therapy options include radiation therapy or surgical management with enucleation. Despite local therapies, uveal melanoma has a high risk of metastatic recurrence, commonly to the liver (93% of metastatic patients), as well as lung (24%), bone (16%), skin or subcutaneous tissue (11%), and lymph nodes (11%) [2, 3]. The median time to develop liver metastases is 2–3 years [4]. Approximately 30%–50% of patients with primary UM will develop metastatic recurrence at 5 years [5]. Retrospective data regarding long‐term prognosis demonstrate that by 15 years, the risk of metastasis is 50%, and further increases to 62% by 35 years [6]. Consequently, long‐term prognosis is guarded in UM patients following treatment of primary disease [7]. Whilst the risk of recurrence continues to climb until 10 years after initial diagnosis [6], the annual risk of systemic recurrence after 10 years ranges from 0.98% to 6.7%, and recurrences after 15 years post‐diagnosis are rare [8]. Conditional 10‐year metastasis‐free survival (MFS), having survived 9 years post‐diagnosis, is 98% [9].

Prognostic factors for recurrence of UM include clinical and molecular features of the primary disease. Clinically, poor prognostic factors are increased tumour size (greater thickness and diameter), location (ciliary body versus others), and histological characteristics (the presence of connective tissue loops, high mitotic count, epithelioid cells) [10]. In a cohort study, the 10‐year metastatic rate for small UMs (0–3 mm thick) was 11.5%, for medium thickness (3.1–8 mm), 25.5% and for large (> 8 mm), 49.2% [11]. American Joint Committee on Cancer (AJCC) stage‐specific survival rates range from 96% to 97% for stage I disease to 25% for stage IIIC [12]. Molecularly, cytogenetic features such as monosomy 3 and chromosome 8q gain and BAP1 loss are associated with poor prognosis and higher risk of metastatic disease, with disomy 3, chromosome 6p gain and EIF1AX mutations portending a better prognosis [13]. Various tools such as the 15‐gene expression profile (GEP) test, The Cancer Atlas Genome (TCGA) Classification, and the Liverpool Uveal Melanoma Prognosticator Online (LUMPO) tool are available for risk stratification and have been externally validated [14].

Effective treatments for metastatic uveal melanoma are limited, with tebentafusp being the only therapy to show an overall survival benefit in metastatic disease in a randomised phase 3 study [15]. However, tebentafusp can only be used in patients who are HLA‐A*02:01. Other systemic therapy options include combination ipilimumab and nivolumab, which showed benefit in the phase 2 GEM‐1402 study [16], and clinical trials such as those testing darovasertib and crizotinib in the metastatic setting (NCT03947385, NCT05987332). In some cases, liver‐directed therapies such as radiation, resection, embolisation, and drug and device combinations such as melphalan hepatic delivery systems [17, 18] are used. More recently, trials have been exploring the role of neoadjuvant and adjuvant therapies, such as the phase 2 investigator‐initiated NADOM trial of neoadjuvant and adjuvant darovasertib [19].

Historically, the paucity of effective therapies for metastatic UM cast doubt upon the need for regular surveillance, with literature showing no impacts on overall survival [20]. Nevertheless, in light of the changing landscape of UM treatment, surveillance and early detection of recurrence may allow early access to systemic therapies. Increasingly, patients with localised UM are being referred to medical oncology services for coordination of surveillance. However, there are currently no standardised Australian guidelines for surveillance imaging in UM following definitive local therapies. Other institutions worldwide have published consensus guidelines, demonstrating variation in practice. As such, there is a need to ascertain the optimal surveillance approach. This in turn may aid the development of national guidelines and influence access to reimbursement. In this systematic review, we examined the literature on surveillance protocols in UM.

## 2 Methods

The study meets the requirements of the Preferred Reporting Items for Systematic Reviews and Meta‐analyses (PRISMA) statement (PROSPERO REGISTRATION NUMBER: CRD42024562864).

### 2.1 ELIGIBILTIY Criteria

Observational studies eligible for this systemic review included cohort studies, retrospective and prospective studies, and case–control studies. Studies were included if they involved adult patients with localised UM who had completed primary therapy, explored the role of surveillance imaging, and reported local and/or distant recurrence rates or information regarding sensitivity or specificity. Studies were excluded if they involved patients with metastatic UM, focused on the restaging of established metastatic disease, or involved non‐uveal melanoma.

### 2.2 Search Strategy

Medline, Embase and PubMed databases were searched using keywords related to uveal melanoma and surveillance (“neoplasm, uveal OR uveal melanoma”) AND (“surveillance OR practice guideline OR screening”). The search strategy was limited to publications in English, and a time restriction was applied from 2010 to the current. Two reviewers (FZ and MA) developed the search strategy, which was conducted in July 2024.

### 2.3 Study Selection

Eligible studies were determined following initial screening and removal of duplicates. Two independent reviewers (FZ and AG) reviewed all studies. In the first stage, title and abstract screening was undertaken—if the eligibility of the study could not be determined, the full text of the article was reviewed. After title and abstract screening, full text articles of remaining records were also screened. References of included studies were also reviewed for potentially relevant articles. Discordance between the independent reviewers was resolved through discussion and oversight by a third reviewer (MA).

### 2.4 Data Extraction

Data extracted from studies included study details (year of publication, study design, journal, sample size, study period), patient details (demographics, stage of diagnosis, risk group), surveillance methodology, test accuracy (sensitivity and specificity), and clinical outcomes such as disease‐free survival (DFS), progression‐free survival (PFS), and overall survival (OS) if reported.

### 2.5 Evaluation of Quality of Studies

The quality of studies was assessed using the modified Newcastle‐Ottawa Scale (mNOS) for cohort studies [21]. The certainty of the evidence, considering risk of bias, inconsistency, indirectness, imprecision, and publication bias, was assessed using the GRADE (Grading of Recommendations, Assessment, Development, and Evaluation) approach [22].

### 2.6 Data Analysis

Due to the heterogeneity of studies and outcomes, a meta‐analysis was not undertaken. Descriptive analysis and synthesis were undertaken, and a narrative review of selected studies was developed.

## 3 Results

### 3.1 Study Selection

**FIGURE 1:**
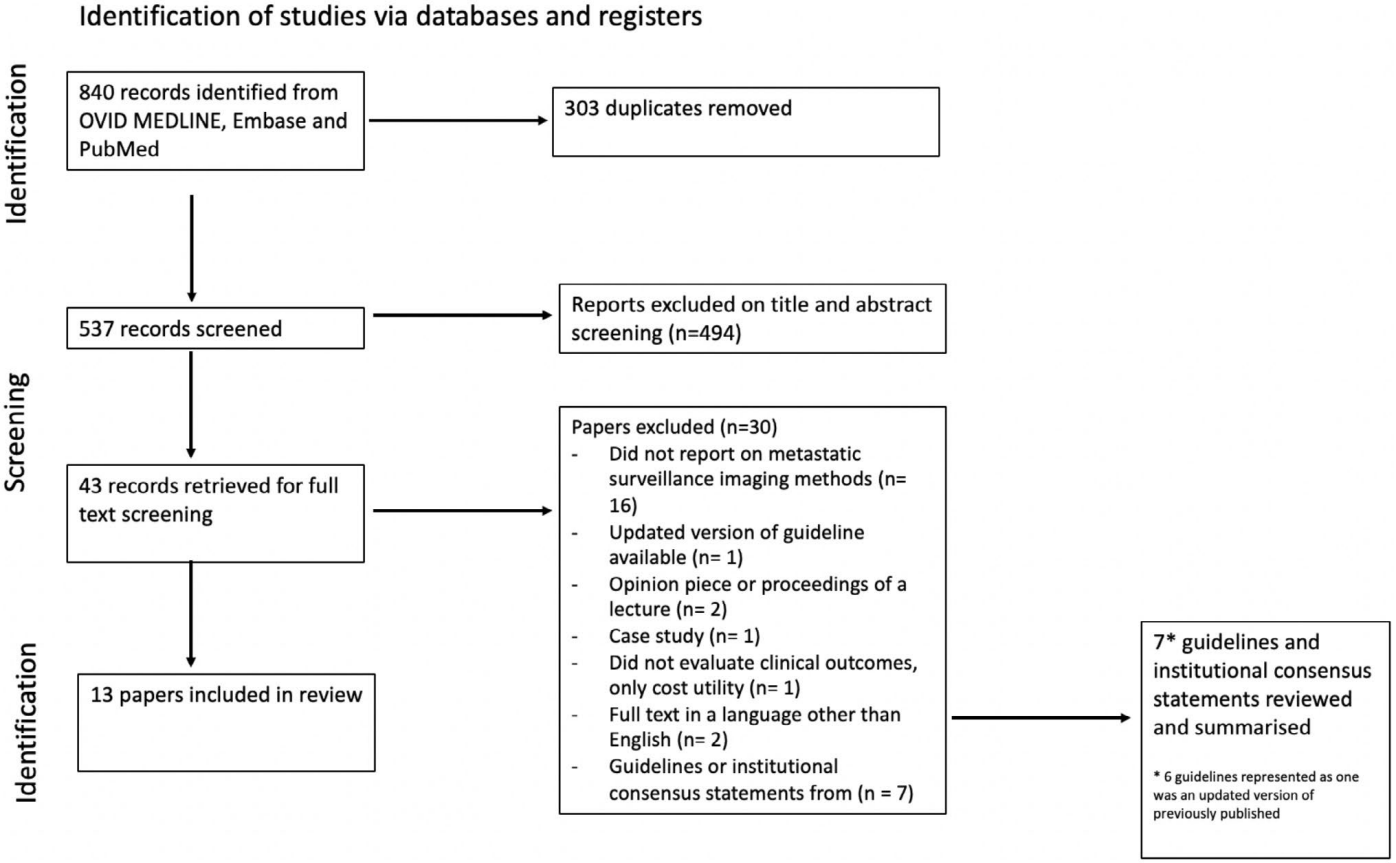
PRISMA flow diagram.

The literature search identified 840 records, from which 303 duplicates were removed (Figure 1). The remaining 537 titles and abstracts were screened. Of these, 404 records were excluded as they were irrelevant to the study question. Full‐text reviews were undertaken of the remaining 43 records, and 30 records were excluded. This included seven guidelines and institutional consensus statements which were excluded to be summarised separately. Of the rest of the excluded papers, the majority [23] did not report on metastatic surveillance imaging methods, one was excluded due to an updated version of the same paper being available, two were opinion pieces or proceedings of a lecture, one was a case study, one did not evaluate clinical outcomes, and two had full texts in languages other than English (French, German). Thus, 13 papers were included in the review.

### 3.2 Characteristics of Studies Included

The 13 studies identified were published between 2012 and 2023 [4, 23, 24, 25, 26, 27, 28, 29, 30, 31, 32, 33, 34]. Of these, seven were cohort studies and two case–control studies involving patients with localised UM. Other studies were reliability analyses and comparisons of risk stratification guidelines. Two studies assessed institutional use of specific surveillance regimens; these will be further explored in the narrative review.

Generally, the evidence in support of a particular surveillance strategy in the literature was poor (Table 1). The quality of the studies was assessed according to mNOS scoring [21], which was chosen as a validated tool to evaluate selection, comparability, and outcomes of non‐randomised studies (Table 2). All studies were assessed as poor quality except Yesitlas et al., which was rated as fair.

**TABLE 1:** Summary of selected studies exploring surveillance strategies.

Author (year)	Study design	Study period	Study population	Modality of surveillance	Frequency/duration of surveillance	Surveillance accuracy	Clinical outcomes
Chopra [24]	Population based cohort study	2014–2016	*N* = 101 Localised UM, all risk groups	PET/CT correlation with liver biopsy	NS	PPV 100%	Distant recurrence rates 5.0%
Choudhary [23]	Retrospective cohort study	Oct 2003—Oct 2012	*N* = 263 Localised UM, all risk groups	CT CAP at baseline, USS Liver and LFTs	6‐monthly for 5 years, then 12‐monthly to 10 years	Sensitivity 96% [80%–99%] Specificity 88% [83%–91%] PPV 45% [33%–59%]	
Choudhary [25]	Retrospective chart review	2003–2011	*N* = 265 Localised UM	CT CAP at baseline, USS Liver and LFTs	6‐monthly for 5 years, then 12‐monthly to 10 years	PPV 53%	Local recurrence rate 4.7%
Lee [27]	Prospective cohort study	April 2008—April 2018, median follow up 46.8 months	*N* = 169	CT, MRI or USS abdomen	6‐monthly for 5 years, then 12‐monthly to10 years	Specificity 78%	Distant recurrence rate 19%—of these patients, 44% relapsed within < 2 years, 44% > 2 to < 5 years, 12% > 5 years from local therapy mPFS 27.4 m mOS 13.5 m
Marshall [26]	Prospective cohort study	Jan 2000—Nov 2010, median follow up 28.8 months	*N* = 188 Median basal diameter 16.5 mm, majority of pts. had Ch3 loss	MRI Liver and LFTs	6‐monthly for 5 years	48% of scans were positive for liver metastases	mPFS 33 m
Mouriaux [28]	Case–control study	Median follow up 53.3 months	*N* = 262	LFTs (AST, ALT, GGT, ALP, LDH)	6‐monthly for 3 years	Sensitivity 12.5%–58.0% Specificity > 90% PPV 9.4%–38.6% NPV > 90%	NS
Rantala [29]	Reliability analysis	Jan 1999—Dec 2016	*N* = 215	USS Liver compared to CT/MRI Liver	For TNM stage I‐II: 12‐monthly For TNM stage III: 6‐monthly	Sensitivity 96% [92%–98%]	NS
Robinson* [30]	Comparison of risk stratification scores	2007–2016	*N* = 1047 UM (choroidal melanoma) post primary therapy	Comparison of LUMPOIII, LPM, AJCC and Monosomy 3 risk stratification scores	Up to 5 years studied	NS	NS
Rola [4]	Retrospective study	2008–2018, median follow up 5.1 years	*N* = 615 Stage I‐III UM Ch3 loss (60%), Ch6p gain (32%), Ch8q gain (59%)	MRI Liver (63%), USS or CT Liver (37%)	NS	24% of pts. had metastases detected on imaging	Distant recurrence—87% within 5 years, 97% within 10 years
Schefler* [31]	Prospective multicentre study of specialty referrals and surveillance regimens used		*N* = 140 Stage I‐III GEP Class 1A 52%, Class 1B 15%, Class 2 33%	Imaging and LFTs	Low intensity—imaging/LFTs 6–12 monthly High intensity—imaging/LFTs 3–4 monthly	NS	Referred for medical oncology follow up 93% (class 2), 51% (class 1) 62% of Class 2 pts. had high‐intensity surveillance 85% of Class 1 pts. had low‐intensity surveillance
Steeb* [34]	Questionnaire of HCPs to evaluate what imaging modality and frequency was used	2018	*N* = 460 Stage I‐III	USS Liver 76.3%, MRI Liver 52.6%, CT 47.4%, PET 21.1%	3‐monthly 66.7%, 6‐monthly 51.5%, 12‐monthly 9.1%	N/A	N/A
Xu [32]	Retrospective observational study	2003–2016	*N* = 73	USS Liver	NS	Average time to liver met 32 ± 39 (SD) months 67% of met detected on routine surveillance USS Liver	N/A
Yestilas* [33]	Case–control study of standard versus enhanced surveillance	2013–2021 Median follow up 36.3 months	*N* = 87 GEP Class 2	Standard protocol: USS Liver Enhanced modality/enhanced protocol: CT/MRI Liver	Standard frequency: 6‐monthly High frequency/enhanced protocol: 3‐monthly	No OS difference between protocols HR for death reduced with EP HR 0.25 [0.07–0.84] USS HR 0.23 [0.06–0.84], EM HR 0.11 [0.03–0.5], compared to standard	N/A

Abbreviations: CT, computed tomography; GEP, gene expression profiling; LFTs, liver function tests; mOS, median overall survival; mPFS, median progression free survival; MRI, magnetic resonance imaging; MRI + c, MRI with contrast; OS, overall survival; PET, positron emission tomography; PFS, progression free survival; SD, standard deviation; USS, ultrasound.

* Studies selected for narrative review.

**TABLE 2:** Assessment of quality of studies according to modified Newcastle‐Ottawa Scale.

Author	Year	Selection				Comparability	Outcome			Quality of assessment
		Representativeness	Selection	Ascertainment	Demonstration	Comparability	Assessment of outcome	Follow up duration	Follow up adequacy	
Chopra	2023	*	0	*	*	0	*	0	*	Poor
Choudhary	2016	*	0	*	*	0	*	0	*	Poor
Choudhary	2013	*	0	*	*	0	*	*	*	Poor
Lee	2018	*	0	*	*	0	*	*	*	Poor
Marshall	2012	*	*	*	*	0	*	*	*	Poor
Mouriaux	2012	*	*	*	*	0	*	*	*	Poor
Rantala	2020	*	*	*	*	0	*	*	*	Poor
Robinson	2023	*	0	*	0	0	*	*	0	Poor
Rola	2022	*	0	*	0	0	*	*	0	Poor
Schefler	2020	*	0	0	0	*	0	0	0	Poor
Steeb	2021	0	0	*	0	0	*	0	0	Poor
Xu	2017	0	0	*	0	0	*	0	0	Poor
Yesiltas	2023	*	*	*	*	*	*	*	*	Fair

Described surveillance strategies for UM consisted of clinical reviews, biochemical testing, and imaging, either alone or in combination. 11 of the 13 studies involved imaging. Two studies assessed the efficacy and accuracy of imaging modalities. Chopra et al. assessed rates of correlation between positron emission tomography (PET)/computed tomography (CT) findings and liver biopsy in a population‐based cohort study of 101 patients with localised UM, spanning all risk groups. They demonstrated that imaging with PET/CT for liver metastases had a positive predictive value (PPV) of 100%, supporting the role of this modality to identify metastatic disease [24]. Rantala et al. undertook a reliability analysis in 215 patients with localised UM, comparing ultrasound (USS) liver with confirmatory CT or magnetic resonance imaging (MRI) liver (every 6 months in Stage III disease and every 12 months in Stage I–II disease). This study found that USS was consistent with CT or MRI in 53% of patients, and that 29% of CT or MRI scans showed more metastases than USS. Overall, the sensitivity of USS in detecting metastases was 96% [92%–98%] [29]. Across the studies, USS was the most frequently used modality across eight studies, with MRI also being assessed in six studies, predominantly in high‐risk populations. Only one study (Mouriaux et al.) investigated the accuracy of surveillance compared with LFT blood test alone (AST, ALT, GGT, ALP, and LDH), undertaken every 6 months for 3 years. Whilst abnormal LFTs for hepatic metastatic disease demonstrated a high specificity of > 90%, sensitivity was low [28]. In contrast, sensitivity across imaging modalities in numerous studies was significantly higher, suggesting that imaging is a key part of any surveillance strategy.

The frequency and duration of surveillance is an important question. Most studies undertook surveillance imaging every 6 months for the first 5 years after diagnosis, and then annually for up to 10 years [23, 25, 26, 27]. This frequency and duration of surveillance is supported by clinical outcome data reported in some papers. For example, Lee et al. [27] demonstrated a distance recurrence rate of 19% overall, with 88% of these recurrences occurring within 5 years after primary diagnosis. Similarly, Rola et al. found that 87% of patients with distant recurrence developed this within 5 years, and 97% within 10 years [4]. Surveillance strategies could be subdivided into standard and enhanced protocols based on frequency and modality, further explored below.

### 3.3 Narrative Review of Select Studies

#### 3.3.1 Yesitlas (2023)

Yesitlas et al. undertook a retrospective case–control study of standard versus enhanced surveillance in 87 patients at high risk of developing UM metastases, based on GEP Class 2 scores. Patients were identified between 2013 and 2021 and included if they had normal baseline staging imaging with CT CAP within 6 weeks of their primary UM treatment, followed by periodic surveillance imaging. The scan rate was calculated from the number of surveillance studies from staging to the end of follow‐up, divided by the number of months of follow‐up. Patients with ≤ 1 image per 6‐month period were defined as standard frequency, and those with a scan rate of > 1 per 6 months were categorised as high frequency. Patients who had a CT or MRI were categorised as enhanced modality (EM), and those who had other modalities such as USS were categorised as standard modality. Patients followed with either high frequency or EM surveillance were then defined as having undergone surveillance with an enhanced protocol (EP). Notably, this study was the only one to be rated fair by mNOS criteria, scoring for patient selection, representativeness of population (patients with high‐risk disease), comparability and assessment of outcome [33].

EP, whether with high frequency or enhanced modalities, detected more smaller metastatic lesions than standard protocols. Patients on surveillance with an EP had a lower 24‐month cumulative incidence of > 3 cm metastasis, and those on high frequency protocols had a higher 24‐month cumulative incidence of ≤ 3 cm metastasis compared to standard protocol. The risk of death following metastasis was significantly reduced with EP (HR 0.25, 95% CI 0.07–0.84), as well as with high frequency (HR 0.23, 95% CI 0.06–0.84) and EM (HR 0.11, 95% CI 0.02–0.5) protocols compared to standard protocols. Taken together, this suggests that modalities such as CT or MRI, as used in the EM protocol, and higher frequency of imaging are associated with better clinical outcomes in patients with high risk UM. In this study, HF and EM protocols were not able to be directly compared due to some patients being included in both cohorts. An unanswered question remains as to whether increased frequency of scans with standard imaging modalities can abrogate the need for enhanced modalities, or vice versa—which would be helpful in guiding surveillance regimen options.

Overall, there was no significant difference in OS between the protocols. The authors postulated that the lack of OS benefit with enhanced protocols may be due to the small number of patients undergoing further local or systemic therapies. Indeed, the study was undertaken prior to the widespread availability of tebentafusp, and the lack of OS benefit may be reflective of the poor prognosis associated with hepatic metastases in UM, in the absence of effective therapy, regardless of early detection. Given only patients with GEP class 2 scores were included, the results cannot be extrapolated to lower‐risk UM patients.

#### 3.3.2 Robinson (2023)

This study involved 1047 patients with choroidal melanoma who underwent standard primary therapy at the Liverpool Ocular Oncology Centre, between 2007 and 2016, and had at least 5 years of follow‐up. Four different prognostic systems were compared with regard to risk stratification for surveillance: the Liverpool Uveal Melanoma Prognosticator Online III (LUMPOIII), the Liverpool Parsimonious Model (LPM), the AJCC staging system, and the use of monosomy 3 as a single marker. Thresholds from low‐risk (no surveillance) to high‐risk (surveillance) categories were set. The defined endpoint for sensitivity and specificity analysis was death from or detection of UM metastasis within 5 years of primary treatment. For each system, patients correctly classified as high risk were considered true positives, and those correctly classified as low risk were considered true negatives. Sensitivity and specificity were calculated using receiver operating characteristic curves (ROC) [30].

Overall, the study demonstrated the thresholds for LUMPOIII or LPM that offered greater specificity at equal levels of sensitivity as compared to AJCC or monosomy 3 systems. The use of LUMPOIII with a threshold of ≥ 0.05 (patients scoring higher than 0.05 being enrolled in the surveillance programme) led to better specificity for the same high sensitivity achieved with the AJCC classification system. Similarly, using LUMPOIII with a threshold of ≥ 0.1 achieved increased specificity for equal sensitivity with the monosomy 3 system. LUMPOIII also offered superior sensitivity and specificity over AJCC in the absence of genetic information, which is applicable to centres without capacity for routine genetic testing or in patient cases where this is not possible. However, whilst the study compared relative sensitivity and specificity of different prognostic systems, it did not suggest optimal levels of sensitivity and specificity that would require further economic analysis. Although the focus of this paper was the evaluation of prognostic systems rather than methods of surveillance, it highlights the importance of risk stratification strategies in any surveillance regimen.

#### 3.3.3 Schefler (2020) and Steeb (2011)

Two papers studied current patterns of referral and practice regarding surveillance and follow‐up in their centres. In Schefler et al. nine oncology centres prospectively enrolled 138 patients with localised UM who were clinically tested with the 15‐GEP score [31]. Data on physician‐recommended specialty referrals and surveillance regimens were collected. Of high‐risk class 2 patients, 93% were referred to medical oncology for follow‐up, compared with 51% of class 1 patients. Most (62%) class 2 patients were recommended for overall high‐intensity metastatic surveillance (with imaging and LFTs every 3–4 months), while 85% of class 1 patients were recommended for low‐intensity metastatic surveillance (with imaging and LFTs every 6 to 12 months). Steeb et al. administered a questionnaire to ascertain patterns of care and surveillance in the management of patients with uveal melanoma across 70 skin cancer centres in Austria, Germany and Switzerland. The frequency distributions of responses for each question were calculated [34]. Most patients with localised UM were referred to skin cancer centres by ophthalmologists (87.2%). Notably, 35.1% of centres to which patients were referred did not perform any screening measures. Approximately two‐thirds of patients underwent imaging every 3 months, and just over half underwent six‐monthly imaging. USS Liver was the most frequently used modality (76.3%), followed by MRI liver (52.6%). Overall, significant variations in practice were reported regarding surveillance in localised UM [31, 34].

### 3.4 Comparison of Guidelines and Consensus Statements

Six guideline and consensus statements were summarised, as the seventh was an earlier version of the Updated Cancer Care Alberta Clinical Practice Guideline [35, 36, 37, 38, 39, 40]. These were published between 2013 and 2023 and included guidelines from the UK, Scotland, American Society of Clinical Oncology (ASCO), NCCN, Canada and the Polish Society of Oncology (Table 2). Most guidelines were clear in specifying risk stratification, frequency and modality of imaging and duration of surveillance.

The criteria used for risk stratification varied across guidelines. Notably, the ASCO Educational Book recommendations [37] and the Uveal Melanoma UK National Guidelines [38] did not clearly define high and low risk, pointing at disparity in clinical approaches. The other guidelines used a combination of cytogenetic and/or AJCC staging criteria to define high and low risk, with the Polish Society of Oncology [39] and the NCCN also describing intermediate or medium risk populations.

For high‐risk populations, all guidelines recommended at least six‐monthly imaging, with the Polish Society of Oncology, ASCO and NCCN guidelines suggesting consideration of three‐ to six‐monthly imaging for the first 5 years, followed by 6 to 12‐monthly imaging until 10 years post diagnosis. Most guidelines recommended surveillance imaging for up to 10 years in high‐risk populations. In contrast, the UK National Guidelines take a more conservative approach, recommending a lifelong duration of surveillance with liver‐specific imaging, clinical review and nurse specialist support every 6 months [38]. MRI or USS liver were most recommended for high‐risk patients, with the Scottish guidelines stipulating the use of MRI liver specifically for high‐risk patient groups.

For medium or intermediate risk populations, there was variability in the recommended surveillance frequency. The Scottish guidelines recommend USS liver every 6 months, whilst NCCN and Polish Society of Oncology recommend 6 to 12‐monthly imaging (either MRI or ultrasound liver with or without CT CAP) [35]. In low‐risk populations, the Polish Society of Oncology indicated imaging studies only ‘if indicated’, whilst all other guidelines do recommend imaging on a regular ongoing basis [39]. For the most part, lower risk patients were recommended 6 to 12‐monthly surveillance imaging, for a period of up to 10 years.

## 4 Discussion

The evidence guiding optimal surveillance strategies for patients with definitively treated localised UM is of variable quality and mainly based on data from cohort and population‐based studies that are limited by their heterogeneity in terms of patient population, risk stratification methods, and the modality and frequency of surveillance utilised. Consequently, a meta‐analysis of the data was unable to be undertaken. Of note, there are no randomised controlled trials comparing surveillance strategies in localised UM.

Imaging is an integral part of any surveillance regimen. Clinical reviews or blood tests, whilst potentially useful as adjuncts to imaging, are insufficient when used alone. However, there is a paucity of robust, randomised data directly comparing imaging modalities. Drawing from recommendations made by institutional guidelines, it can be surmised that MRI is a modality with superior sensitivity, compared to USS and CT. Indeed, in general radiology guidelines, MRI liver is the gold standard for non‐invasive diagnosis of liver metastases [41]. Over half of the studies included in this literature review included use of MRI. Notably, MRI liver as a regular surveillance modality is consistently recommended as the preferred modality, at least for patients with high‐risk disease, across the Scottish, Polish and NCCN guidelines. Another merit of MRI in addition to its increased sensitivity is the lack of radiation exposure it confers, which may be preferred in a disease necessitating long‐term surveillance wherein minimising risks of long‐term radiation exposure is important. Nevertheless, there are challenges with MRI including accessibility, cost, and time, which may limit its use to resource‐rich settings. The duration of surveillance in most guidelines and studies was up to 10 years, in line with data showing that most recurrences occur during in this period [35].

Indeed, radiation‐exposure related risks need to be considered. Wen et al. studied the lifetime attributable risk (LAR) of cancer associated with various combinations and frequencies of CT and PET surveillance commonly used in the surveillance of patients with choroidal melanoma. They found that on average, for a patient aged 50 years, annual CT CAP for 10 years is associated with an estimated LAR of cancer of 0.9%–1.3%, and 1.6%–19% with annual PET/CT, with even higher LAR of cancer in younger and female populations [42]. Prognosis with metastatic UM remains guarded. Noting the delay between radiation exposure and development of secondary malignancies, most patients, particularly those with high‐risk UM, are unlikely to survive long enough for this to be a significant risk. On the other hand, the risks of radiation exposure ought to be more carefully considered in younger patients and those with lower risk UM, and may need to be re‐evaluated in the context of better systemic therapies.

Whilst several studies reported on clinical outcomes such as PFS and OS in the cohorts investigated, it is not possible to draw firm conclusions regarding the impact of surveillance on survival outcomes. Yesitlas et al. did not observe significant OS differences when comparing enhanced frequency and standard frequency surveillance imaging. Almost all the studies included were undertaken prior to the widespread use and availability of tebentafusp, the only systemic therapy that has demonstrated OS improvement in metastatic UM and available only to HLA‐A*02:01 patients. Furthermore, subgroup analyses of the IMCgp100‐202 study of tebentafusp in metastatic UM demonstrated that patients with smaller hepatic metastatic lesions (largest metastatic lesions ≤ 3.0 cm) derived greater benefit from tebentafusp than those with larger lesions [43] Similarly, in the phase 3 SCANDIUM trial of isolated hepatic perfusion therapy, treatment benefit was limited to those patients with smaller metastatic lesions [44]. Thus, there may be a clinical benefit to early detection of metastatic disease. In the current landscape of UM management, there is also a need to further explore the cost‐effectiveness of surveillance regimens.

Knowledge of the molecular and cytogenetic prognostic factors in UM has been incorporated into some international guidelines and refined approaches to surveillance. Yet there is scope to develop more personalised risk stratification tools to improve surveillance approaches [45]. Developments in liquid biopsy techniques and biomarker surveillance may also aid in risk stratification and guiding imaging. Prospective data from patients with UM with treated primary tumours demonstrated that circulating tumour DNA (ctDNA) levels correlate with radiological progression and that detection of ctDNA is associated with poorer survival [46]. Moving forward, a more tailored approach to surveillance may improve current practices, patient outcomes, and health resource allocation.

Despite the heterogeneous data in the literature around UM surveillance, numerous institutional guidelines from European and North American societies are frequently cited. It is likely that clinicians globally use these to guide clinical practice. Yet, adherence to such recommendations is variable, as noted in several studies included in this review [31, 34]. (Table 3).

**TABLE 3:** Summary of current institutional guidelines for surveillance of patients with uveal melanoma following local definitive treatment.

Guideline	Author (year)	Risk stratification	Modality	Frequency	Duration
Consensus statement for metastatic surveillance of uveal melanoma in Scotland. [Review] [35]	Chadha (2023)	High Risk—presence of any of: – AJCC (8th Edition) Stage IIIA or worse – Monosomy 3 – Abnormalities is Chromosome 8 (8p loss, 8q gain) – BAP‐1 mutations – In absence of cytogenetics testing, Presence of high‐risk pathological features including epithelioid cells, extra‐scleral extension and presence of closed connective tissue loops—decision to be made at MDT – Any other features of tumour of other factors that may indicate high risk of metastases—decision to be made at MDT	MRI Liver with (+c) and without contrast (−c) MRI Liver −c (CT triple phase liver if MRI contraindicated)	At diagnosis 6‐monhtly	10 years
		Low or Medium‐Risk: – All melanomas not classified as High Risk	USS liver USS liver—if equivocal, limited visualisation or suspicious finding, for MRI liver +c	At diagnosis 6‐monthly	10 years
Surveillance options for patients with uveal melanoma following definitive management. [Review] [37]	Francis (2013)	High Risk	Imaging – MRI: high resolution/sensitivity – PET: Sensitivity 40%–100%, Specificity 67% – CT: High sensitivity, Low PPV – Abdominal USS: Sensitivity 14%, Specificity 100%	3–6 monthly	Not specified, likely up to 10 years
		Lower Risk	Imaging	6–12 monthly	
Uveal Melanoma UK National Guidelines. [Review] [38]	Nathan (2015)	High Risk	Liver‐specific imaging, clinical review, nurse specialist support	6‐monthly	Life long
NCCN Guidelines Insights: Uveal Melanoma, Version 1.2024 [40]		High Risk: – Class 2 GEP – Monosomy 3 – Gain of chromosome 8q – BAP1 mutation – T4 (AJCC)	– Imaging to evaluate signs or symptoms – MRI Liver +c or USS Liver (±CT CAP or CXR)	3–6 monthly 6–12 monthly	5 years Years 6–10
		Medium Risk: – Class 1B GEP – SF3B1 mutation – T2 and T3 (AJCC)		6–12 monthly	10 years
		Low Risk: – Class 1A BEP – Disomy 3 – Gain of chromosome 6p – EIF1AX mutation – T1 (AJCC)		12 monthly	5 years
Diagnostic and therapeutic management of patients with ocular melanomas—recommendations of the Polish Society of Oncology [39]	Rutkowski (2022)	High Risk: – T3 – Known molecular abnormalities—chromosome 3 monosomy, multiple copies of 81, BAP1 mutation, PRAME expression	Imaging – MRI liver +c (preferred) – USS Liver – Additional CT CAP or PET	3–6 monthly 6–12 monthly	5 years Years 6–10
		Intermediate Risk: – T2 or T3 – FJ3B1 mutation	Imaging	6–12 monthly	Not specified
		Low Risk: – T1 – Disomy chromosome 3, multiple copies of 6p, EIF1AX mutation	Imaging studies if indicated	Not specified	Not specified
Management of Uveal Melanoma: Updated Cancer Care Alberta Clinical Practice Guideline. [Review] [36]	Weis (2023)	Higher Risk: – Class 2 GEP – Monosomy 3 – Tumours > 9 mm thick/12 mm maximal basal dimension	Alternating Liver USS and MRI Liver	6 monthly	10 years
		Lower Risk: – Class 1 GEP – Disomy 3 – Tumour ≤ 9 mm thick/12 mm maximal basal dimension	Liver USS	12 monthly	10 years

Abbreviations: AJCC, American Joint Committee on Cancer; CT, computed tomography; GEP, gene expression profiling; MRI, magnetic resonance imaging; MRI + c, MRI with contrast; PET, positron emission tomography; USS, ultrasound.

In conclusion, surveillance has a role following primary therapy in localised UM. Based on the current literature and institutional guidelines, the favoured approach depends on the risk category. MRI is the preferred modality in cases with a high risk of metastatic disease, whereas USS, CT, or an alternating combination of imaging modalities is suitable in medium to low risk disease. With regards to frequency, high risk group patients should have surveillance imaging at least every 3–6 months in the first 5 years, with potential to decrease to 6 to 12 months thereafter. In medium or intermediate risk cases, imaging every 6 to 12 months is reasonable, whilst in low‐risk disease, annual imaging is likely to be sufficient. Imaging beyond 10 years is likely unnecessary. However, the decision as to frequency may come down to individual patient and clinician preferences regarding the risks and benefits of surveillance.

As there are no published Australian guidelines or studies in the literature regarding UM surveillance, it will be important to ascertain real‐world evidence of current practice amongst Australian clinicians in this area. Indeed, some Australian centres are already contributing to global registries such as the OMNi study [47] and the Fight Tumour Blindness Registry [48], which aim in part to address this gap. Ultimately, there is a need for guidelines that consider local nuances, such as the use of molecular testing to classify risk, government rebates for specific imaging modalities, and issues of feasibility, accessibility, and health economic implications.

## Conflicts of Interest

D.K.: Advisory boards—BMS, MSD, Medisons Pharma Australia. A.M.J.: Medison (to Institution); Ideaya (to Institution).

## Data Availability

The data that support the findings of this study are available from the corresponding author upon reasonable request.
